# Food supply and bioenergy production within the global cropland planetary boundary

**DOI:** 10.1371/journal.pone.0194695

**Published:** 2018-03-22

**Authors:** R. C. Henry, K. Engström, S. Olin, P. Alexander, A. Arneth, M. D. A. Rounsevell

**Affiliations:** 1 School of Geosciences, University of Edinburgh, Edinburgh, United Kingdom; 2 Department of Physical Geography and Ecosystem Science, Lund University, Sölvegatan 12, Lund, Sweden; 3 Land Economy and Environment Research, SRUC, Edinburgh, United Kingdom; 4 Karlsruhe Institute of Technology, Institute of Meteorology and Climate Research, Atmospheric Environmental Research (IMK-IFU), Kreuzeckbahnstr. 19, Garmisch-Partenkirchen, Germany; Wageningen University, NETHERLANDS

## Abstract

Supplying food for the anticipated global population of over 9 billion in 2050 under changing climate conditions is one of the major challenges of the 21^st^ century. Agricultural expansion and intensification contributes to global environmental change and risks the long-term sustainability of the planet. It has been proposed that no more than 15% of the global ice-free land surface should be converted to cropland. Bioenergy production for land-based climate mitigation places additional pressure on limited land resources. Here we test normative targets of food supply and bioenergy production within the cropland planetary boundary using a global land-use model. The results suggest supplying the global population with adequate food is possible without cropland expansion exceeding the planetary boundary. Yet this requires an increase in food production, especially in developing countries, as well as a decrease in global crop yield gaps. However, under current assumptions of future food requirements, it was not possible to also produce significant amounts of first generation bioenergy without cropland expansion. These results suggest that meeting food and bioenergy demands within the planetary boundaries would need a shift away from current trends, for example, requiring major change in the demand-side of the food system or advancing biotechnologies.

## 1. Introduction

Projected population growth to over 9 billion people by 2050, the impacts of climate change on agricultural productivity [1–4], and shifts toward animal-based diets [5–8] all pose a challenge to ensuring food security in the future. The proportion of globally undernourished people halved between 1990 and 2015; falling from 23.3% in 1990–92 to 12.9% in 2015[9]. Nonetheless, there were still around 800 million undernourished people in 2015, mostly in Asia and Africa. Achieving food-security in the future will require sufficient food production, as well as its equal distribution and access by all people, as stated in the second UN Sustainable Development Goal [10].

Historically, cropland expansion into forest and other unmanaged natural areas has met increased demand for land-based commodities [11]. Since the mid-20^th^ century, while for the most part agricultural intensification was responsible for increased production, further expansion of agricultural areas still occurred [7,12]. Cropland expansion and intensification both have high environmental costs including GHG emissions, the loss of biodiversity, fresh-water depletion, and environmental pollution from chemical inputs [12].Rockström et al. [13] suggested that global cropland area should be limited to 15% of the total ice-free land surface in order to stay within the planetary boundary for land-use. The planetary boundary approach first identifies earth system processes and thresholds that when crossed may cause undesirable environmental change. Planetary boundaries are defined through normative judgements ensuring values for control variables are kept at an acceptable distance from thresholds [14]. Crop cultivation currently covers about 12% of the global land surface thus the cropland planetary boundary would allow for a 3% expansion in cropland area [12]. A compounding challenge is the mitigation of, as well as adaptation to, climate change [15–17]. Climate mitigation strategies need to consider both the energy and agricultural sectors in order to avoid spill over effects, such as the rapid expansion of cropland arising from bioenergy production [18,19]. Current thinking suggests bioenergy, in combination with carbon capture and storage (BECCS), is an essential strategy for emissions reduction [20–22]. However, expansion of the area used to grow bioenergy will conflict with food production and have potential impacts on biodiversity and the supply of ecosystem services[23].

Taken together there are large pressures on the land system to feed a growing population, to contribute to climate mitigation, and to nonetheless stay within the planetary boundary for cropland area. Over recent decades, scenario analyses have played a central role in assessments of addressing such growing pressures, seeking to explore the potential effects of socioeconomic change and global environmental change. Such scenarios are derived from an interpretation of coherent, qualitative storylines describing future pathways or projections [24–27]. Exploratory scenarios describe different future states that may arise in the absence of setting targets, often extrapolating current trends e.g. in food demand, cropland expansion, and climate [27–29]. For example, recent work using integrated assessment models have explored multiple issues including future climate, land use, and energy sector developments that might occur without considering explicit environmental or energy policies [4,30,29,31–33].

In a normative (or goal-orientated) scenario approach, targets representing a desired situation at some point in the future are first defined and pathways to achieve these targets are interpreted or derived, often from model simulations [34]. Existing normative scenario approaches have defined targets and interpreted pathways to meet food demand [35] environmental constraints [36] and bioenergy targets [37,38]. In some cases more than one target was considered, for example, Erb et al. [36]asssessed the option space for meeting estimated global food demand under different diets in 2050 with low or no levels of deforestation. However, a normative approach that simultaneously considers boundary targets to reducing global food shortages, restrict land use change and meet bioenergy demand has not yet been considered.

In this paper, we explore whether meeting these three multiple objectives is feasible in the future. Unlike similar modelling frameworks the Parsimonious Land-Use Model used here has rapid runtimes and thus allows for multiple model iterations to explore across parameter space within plausible uncertainty ranges [39]. Given this strength, we use a Monte Carlo modelling and normative scenario approach to identify the key characteristics of alternative futures that ensure adequate global food supply by 2050 (food-supply target) whilst maintaining global cropland area within a planetary boundary (cropland planetary boundary target) and, at the same time, producing sufficient bioenergy to contribute to keeping the global average temperature increase below 2°C (bioenergy-mitigation target). Bioenergy deployment has significant potential for climate mitigation, through the reduction of GHG emissions. Modelling work has highlighted that bioenergy could contribute to mitigation despite associated land use change [30]. Creutzig et al. [40] found that in climate stabilisation scenarios across numerous modelling studies between 10 and 245 EJ yr^−1^ of the global primary energy supply in 2050 was supplied by bioenergy. For the bioenergy-mitigation target we therefore take a conservative approach and set the contribution of first generation bioenergy crops to 9 EJ by 2050. This aligns with the bioenergy pathway projected in the world energy outlook [41] 450 scenario with atmospheric GHG concentrations stabilized below 450ppm CO_2_eq, resulting in a 50% likelihood of keeping the global average temperature increase below 2°C compared to pre-industrial levels.

## 2. Methods

### 2.1 Normative targets

The food-supply target should ensure adequate food supply for the entire global population. Dietary requirements were estimated from a global population of 9.1 billion in 2050 [42] and a daily dietary energy requirement of 2350 kcal per capita per day (kcal cap^-1^ d^-1^; [43]). The per capita food consumption statistics [44] used here deduces food waste at the household level. Global average food waste at the household level is estimated to be 12% [45] and so, the food-supply target was set to a minimum of 2635 kcal cap^-1^ d^-1^ on average per country.

The planetary boundary target for global cropland area assumes that up to 15% of the global, ice-free terrestrial surface can be used for crop production [13]; the current area is 12%. Assuming a total ice-free land area of 13400 Mha we set 2010 Mha as the global cropland planetary boundary.

For the bioenergy-mitigation target we chose the bioenergy pathway projected in the 450 scenario from the world energy outlook [41]. Given unknown contribution of second generation energy crops we only modelled first-generation bioenergy crops, which currently contribute 3–4% of global bioenergy production [46]. Recent trends have shown an increasing uptake of bioenergy crops for fuel, for example, the use of modern biomass for liquid and gaseous energy carriers increased by 37% from 2006 to 2009 [46]. We assume that the global contribution of first generation bioenergy crops would double from 2000 to 2050 as countries increasingly move away from using low efficiency traditional biomass such as wood, straw and dung, to higher efficiency energy crops. In the 450 scenario the contribution of bioenergy, traditional and modern biomass, is projected to increase from 10% of global primary energy demand today to 15% by 2035 (2235 million tonnes oil equivalent (Mtoe), which is 94 EJ by 2035 and 125 EJ when extrapolated to 2050; [41]). Thus, we set the bioenergy-mitigation target for first generation bioenergy crops to 9 EJ by 2050.

### 2.2 Assumptions for model simulations

The Parsimonious Land-Use Model was used to simulate the agricultural system until 2050 (detailed descriptions of PLUM can be found in; [39,47]. PLUM simulates agricultural land use change in cereal land as a proxy for changes in cropland with cereal used for food and animal feed demand. The trade mechanism assumes countries have access to a global market. Demand in a country can be met by domestic cereal production and through imports. Countries with production in excess of demand export the surplus. Previous work evaluating PLUM against observation data found at a global scale the range of model results simulated could reproduce consumption, production and agricultural land use patterns [39].

Population, income levels and diet drive the demand for agricultural products as previously described in Engström et al. [39]. Calculations of food demand are dependent on population and economic development and are described by statistical relationships revealed by historical country-level statistics from reported FAOSTAT data [44]. The coefficients characterising these relationships are used as scenario parameters. We assumed that population and economic growth continue along current trends (based on the Shared-Socio-economic Pathway SSP2 “Middle of the road”;[48]. The SSPs are part of a scenario framework, established by the climate change research experts to provide a common basis for the exploration of climate mitigation policies, impacts, adaptation options [27]. FAO projects global food demand to increase by 60–70% in 2050 compared with today, that assumes medium population and economic development [49,50]. Thus adopting values for scenario parameters that are based on SSP2 assumptions of continued growth at current trends aligns with FAO projections and recent academic research. Average nutritional status, per country was represented by daily food supply (kcal cap^-1^ d^-1^, see S1 File for details). Changes in demand and yield, due to technological change, result in agricultural land-use change. The technology change parameters determine how rapidly the yield gap reduces over time. For the parameterisation of socio-economic processes, such as food consumption or crop yield increases due to technological development, parameter settings that reproduce current trends (2000–2010) were defined as given in Table 1. Ranges around these values, indicated by the minimum and maximum values (Table 1), were selected to provide wide, but plausible uncertainty ranges, e.g. average per capita meat consumption in developed countries of 70–121 kg in 2050 (Table 1, class *meat 1*). Parameters held constant and justification for doing so can be found in the S1 Table. Uncertainties in future projections, derived from parameter assumptions, were explored through the analysis of the PLUM parameter space.

**Table 1:** Overview of PLUM parameters and uncertainty explored. Columns 3–5: Parameter setting that reproduces the average current trend, between 2000–2010, and the minimum and maximum settings of the uncertainty range for the parameter of interest. Column 6–8: Implications of current trend values (mean), minimum (min) and maximum values (max) for output in the year 2050 with other parameters held at mean values.

Parameter	Description		PLUM Parameter setting			Resulting outcomes in 2050, with other parameters held at current trend value		
			Current trend	Min of uncertainty range	Max of uncertainty range	Current trend	Min of uncertainty range	Max of uncertainty range
						Average meat/milk consumed per capita (pc)		
meat 1			-30	-50	50	82kg	70 kg	121kg
milk 1			-50	-200	50	268kg	198 kg	327 kg
*Group 1 consumption parameters apply to high income countries with a high per capita consumption of animal products but where the increase has been observed to slow down*								
meat 3			45	0	60	83 kg	36 kg	100 kg
milk 3			45	0	90	162 kg	108 kg	213 kg
*Group 3 consumption parameters apply to countries transitioning from low/moderate consumption of animal products to moderate/high consumption*								
meat 4			15	5	43	40 kg	20kg	90 kg
milk 4			45	20	70	109 kg	61 kg	157 kg
*Group 4 applies to countries that do not yet have the means to increase consumption of animal products rapidly*								
consL (*kg meat/milk per capita/)*	Global limit on the consumption of animal products (e.g. a tax on meat products)		0	-60	20	max 122.5 kg meat consumed pc, no implications for milk	max 62.5kg meat consumed pc, max 180 kg milk consumed pc	max 142.5 kg meat consumed pc, no implications for milk consumption
cerealCon *(1/time)*	Global parameter. Additional cereal consumption within a country		0	0	0.4	-	-	+20% cereals pc in countries with kcalPc_i<2200
						Global demand for cereal feed		
fcr improvement (*1/time*)	The effect of technology change on animal production efficiency (feed conversion rate efficiency)		0.2	0	0.2	1461Mt	1623Mt	1461Mt
feedRatio Cap (*1/time)*	Maximum proportion of meet produced using feed (cereals).		0	0	0.2	1461Mt	1461 Mt	2037 Mt
						Global average yield of		
technology (*1/time*)	Yield increases with changing technological development		1.7	0	2	5.13 ton ha^-1^	4.12 ton ha^-1^	5.24 ton ha^-1^
investments *(1/time)*	Yield increases as a function of GDP per capita		1.7	0	1.7	5.13 ton ha^-1^	5.04 ton ha^-1^	5.14 ton ha^-1^
residualNV *(%)*	Minimum global area of natural vegetation to be kept		10	1	20	Influences distribution of cropland across countries, no clear direction of impact on global scale		
						Global cereal production loss		
croplandDeg *(1/time)*	Global share of lost production due to cropland degradation Influences the production capacity of cereal land		0.05	0.04	0.1	2.5%	2%	5%
shareBEcr* *(%)*	Contribution of first generation bioenergy crops to global bioenergy production		4 or 12	-	-	4: 3.5% of bioenergy are produced from energy crops in 2050		
						12: 7% of bioenergy are produced from energy crops in 2050		

### 2.3 Identifying model parameterisations that achieve the normative targets

Using the parameter values from Table 1, we performed 120 000 Monte Carlo model runs divided into two sets. For the first 60 000 runs, we assumed bioenergy production to be as projected by the Current Policy scenario (extrapolated to 74 EJ bioenergy by 2050;[41]), and a contribution of first generation bioenergy crops to total bioenergy production of 3.5% by 2050 (3 EJ). For the second 60 000 runs, we used the bioenergy production projected in the 450 scenario (extrapolated to 125 EJ by 2050; [41]) and a contribution of first generation bioenergy crops to total bioenergy production of 7% by 2050 (9 EJ). The minimum and maximum parameter values from Table 1 were used to create uniform probability distributions for each parameter. Parameter sampling was performed by applying a stratified Sobol sequence [51]. Unlike tradition ‘one at a time’ methods that only sample a subset of parameter space and emphasise the central point the Monte Carlo approach using the Sobol sequence sampling method can generate parameterisations that systematically explore joint parameter uncertainty space. Furthermore the sampled values are selected with respect to previously sampled values this avoids sample clustering or gaps and thus reduces sampling discrepancies [51]. The number of simulation runs was chosen as a trade-off between maximising the number of simulations within the time constraints of undertaking multiple model runs.

Crop yield inputs to PLUM were simulated with the global dynamic vegetation model LPJ-GUESS [52,53] (see[47] for implementation details). LPJ-GUESS simulates the biophysical response of crop yields to changing environmental conditions, such as changes in temperature and precipitation patterns, and the CO_2_ fertilization effect. To ensure consistency between the energy scenarios underlying the bioenergy projections and the climate scenarios underlying the crop yield projections, different yield projections were used for the first and second set of runs. For the first set of runs we assumed higher levels of climate change and so, simulated crop yields based on climate data for RCP6.0 [54]. For the second set, which assumed stabilization of greenhouse gases at 450 CO_2_eq, we simulated crop yields based on the more stringent climate mitigation scenario, RCP2.6 [55].

The simulation runs were analysed to identify those model parameterisations (i.e. individual model runs) that fulfilled: a) the food-supply target, b) both the food-supply and cropland planetary boundary targets and c) all three targets: the food-supply, cropland planetary boundary, and bioenergy-mitigation target. From these individual model parameterisations, we created output narratives to describe the key elements of the model runs that were instrumental in achieving the normative targets.

## 3. Results and discussion

### 3.1 Meeting the food supply and cropland planetary boundary targets

**Fig 1:**
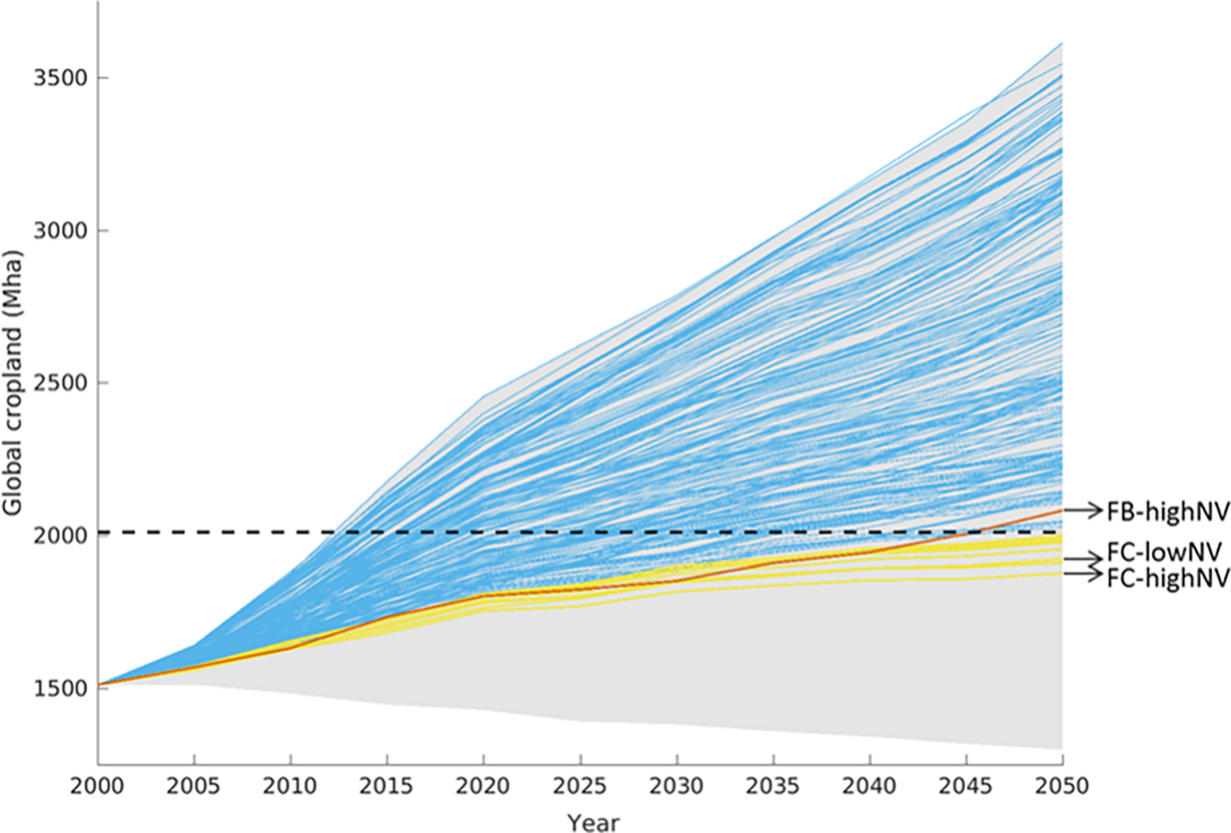
Global cropland area for simulation runs that meet normative targets. Global cropland area for simulations that meet the food-supply target (blue lines) and which are also below the planetary boundary for cropland area (yellow lines, planetary boundary for cropland illustrated by the black dashed line). The red line indicates the run that is closest to meeting all three targets, including the bioenergy-mitigation target (Food-Bioenergy-high-Natural-Vegetation). The grey shaded area indicates the range spanned by all runs. The acronyms shown are discussed in section 3.2.

**Fig 2:**
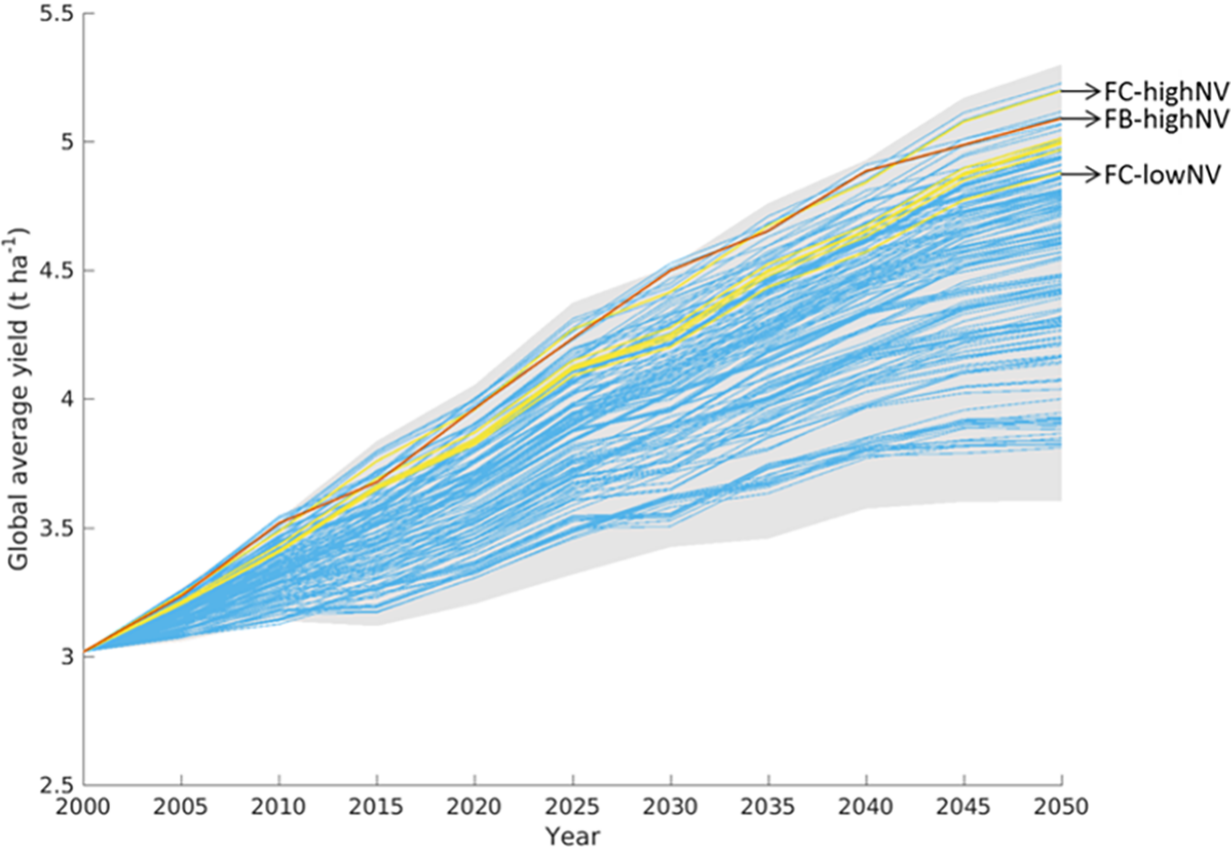
Global average yields for simulation runs that meet normative targets. Global average yields for simulations that meet the food-supply target (blue lines), and which are also below the planetary boundary for cropland area (yellow lines). The red line indicates the run that is closest to meeting all three targets, including the bioenergy-mitigation target (Food-Bioenergy-high-Natural-Vegetation). The grey shaded area indicates the range spanned by all simulations. The acronyms shown are discussed in section 3.2.

The food-supply target alone can be met by a large number of different simulations (Fig 1, blue lines, see also S1 Fig), but most of these simulations require global cropland expansion beyond the 15% planetary boundary by 2050. When the cropland planetary boundary target is also considered, the number of simulations drops sharply to only 12 (Fig 1, yellow lines). Simulations achieving both the food-supply and cropland planetary boundary target have global average yields that are within the upper third of projected yield increases (Fig 2, yellow lines), with yield growth rates of 0.9 to 1.0% yr^-1^ over the 2000–2050 period.

### 3.2 Meeting all three targets: The food-supply, cropland planetary boundary, and bioenergy-mitigation target

**Fig 3:**
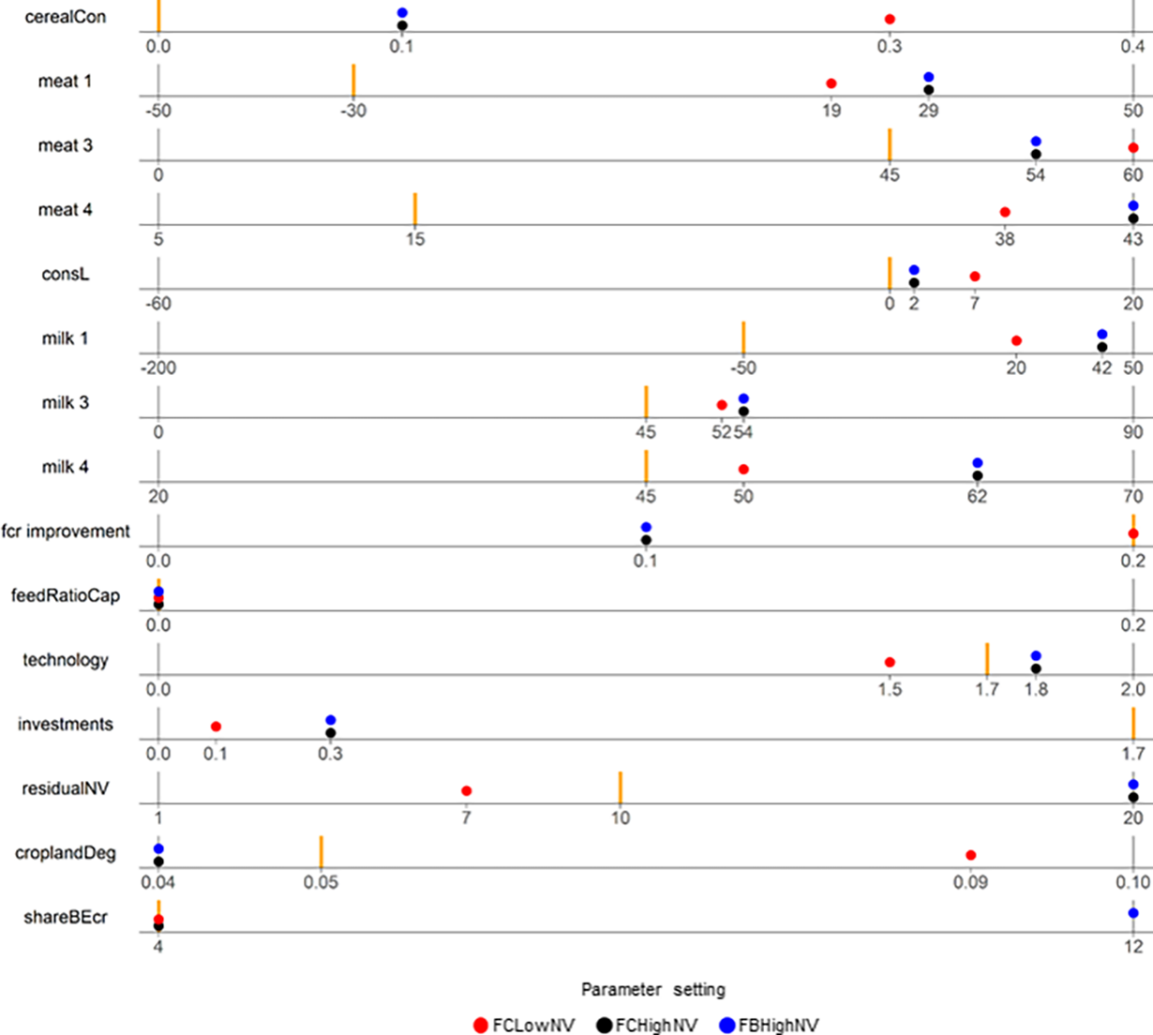
Parameter settings for the three scenarios that almost meet normative targets. The minimum and maximum of the parameter range is shown, with the parameter value that would reproduce current trends (2000–2010) indicated in orange.

Although no model runs achieved all normative targets, three model parameterisations came close to this. Of these, two model runs, named FC-lowNV and FC-highNV, achieve both the food-supply and cropland planetary boundary targets, but not the bioenergy-mitigation target. The third model run, named FB-highNV, fulfils the bioenergy target however, global cropland expansion surpasses the cropland planetary boundary target by 2050. Parameter settings for the three scenarios can be found in Fig 3 and descriptions of the three scenarios are as follows:

#### FC-lowNV (Food-Cropland-low-Natural-Vegetation)

In *FC-lowNV* only 7% of potential arable land is conserved as natural vegetation at a country level (*residualNV*, see Fig 3). Consumption of cereals in countries with low initial food supply increases significantly and per capita meat consumption, especially in transitioning countries, also increases strongly (*cerealCon* = 0.3 and *meat 3 =* 60, see Fig 3). Efficiency in livestock production increases i.e. feed-conversion ratios increase (*fcr improvement = 0*.*2*, Fig 3) and crop yields increase at a moderate to high level (*technology = 1*.*5*, Fig 3).

#### FC-highNV (Food-Cropland-high-Natural-Vegetation)

In contrast to *FC-lowNV*, in *FC-highNV*, 20% of potential arable land is conserved as natural vegetation at the country level (*residualNV*, Fig 3). Per capita cereal consumption in countries with low initial food supply increases slowly and developing countries increase their per capita consumption of meat and milk at very high rates (*meat 4* and *milk 4*, Fig 3). Efficiency in livestock production increases moderately, but crop yields increase at high to very high levels (*technology*, Fig 3).

#### FB-highNV (Food-Bioenergy-high-Natural-Vegetation)

*FB-highNV* has exactly the same parameterisation for consumption, technological change, and protection of natural vegetation as the *FC-highNV* parameterisation, but differs by fulfilling the bioenergy target (*shareBEcr = 12)*. However, this is only achieved by global cropland expansion surpassing the cropland planetary boundary target by 2050. The global cropland area is 2082 Mha in 2050, which is 15.5% of the total ice-free land area (Fig 1, red line). That is to say, this parameterisation is only 0.5% beyond the assumed planetary boundary for global cropland area.

In general, meeting the food-supply target required a strong increase in meat and milk consumption in emerging and developing countries (82–97 and 75–97 kg meat per capita in 2050, from 51 and 22 kg meat per capita in 2000 respectively) and thus a continuation/expansion of Western diets. Only small differences with respect to consumption patterns exist between FC-lowNV and FC-highNV. In 2050, simulated average meat consumption in developing countries in FC-lowNV is 10 kg meat per capita lower than the average meat consumption in FC-highNV. The lower consumption of animal products in FC-lowNV is balanced by an increase in cereal consumption for countries with very low initial food supply (< 2200 kcal cap^-1^ d^-1^ in 2000) in FC-lowNV. For FC-lowNV the increase in cereal consumption for countries with low initial energy supply is 13% by 2050 compared to 2000, while it is 6% for FC-highNV. In PLUM, milk and meat consumption are proxies for rising incomes shifts to protein-rich, high-fat diets. This relationship is well established in the literature [5], but saturation effects (i.e. levelling out of meat consumption, as described by Engel’s law, with slightly different saturation levels dependent on cultural contexts) are assumed to occur in the future [50]. None of our simulations with lower meat consumption (e.g. 70 kg meat per capita in 2050 for countries with traditionally high meat) met the 2050 food-supply target. The target could only be reached when all countries adopted higher calorie, meat-rich diets, resulting in higher annual growth rates in meat consumption (2.9%, 970 Mt meat in 2050) compared to current FAO statistics (2.1% for 2000–2009) or FAO projections (1.4%, 455 Mt meat in 2050;[50]).

**Fig 4:**
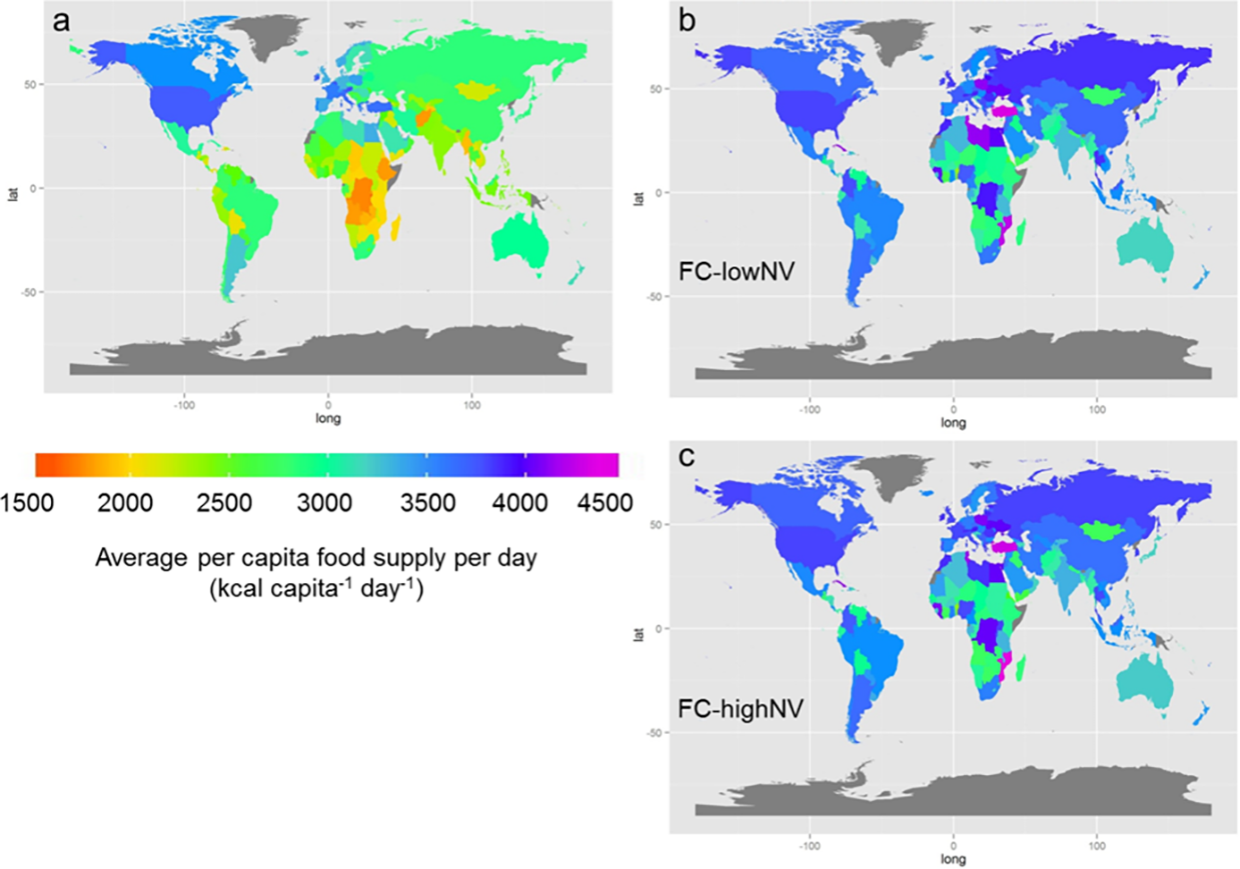
Average daily food supply per capita for the three focal scenarios. (A) Average daily food energy supply per capita in the baseline year 2000 and in 2050 for (B) FC-lowNV and (C) FC-highNV. Countries displayed in grey are excluded from the analysis due to missing input data.

**Fig 5:**
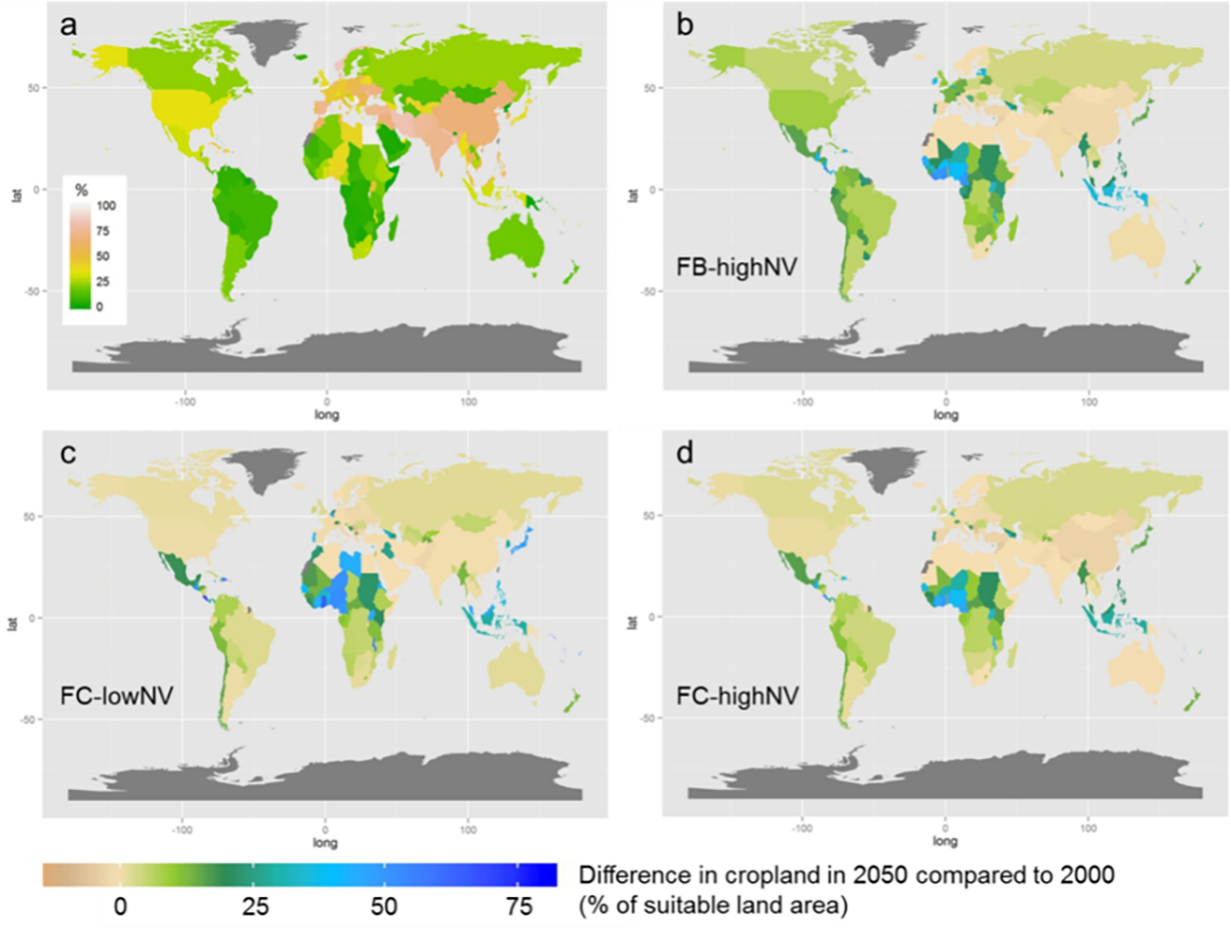
Global cropland area changes over time for the three focal scenarios. (A) Per country cropland area as a percentage of suitable land (moderate to very high suitability from the Global Agro-ecological Zone Data Portal; (FAO/IIASA, 2011). Changes in cropland area in 2050 compared to 2000 (% of suitable land) for (B) FB-highNV, (C) FC-lowNV and (D) FC-highNV. Countries displayed in grey are excluded from the analysis due to missing input data.

The differences in food consumption parameters in FC-lowNV and FC-highNV did not lead to large differences in the average food supply per capita at the country level by 2050 (see Fig 4, panel b and c). For both parameterisations, the surplus food supply observed in 2000 for western countries has spread across most parts of the world by 2050. While food supply is still generally lower compared to western countries, the food shortages in mainly Sub-Saharan African (SSA) countries have been eliminated (indicated by the orange colours in Fig 4, panel a). This increase in food supply corresponds to increasing cropland in SSA (Fig 5). However the ability to improve agricultural production in SSA remains unclear and it has been suggested that the expansion of cropland in SSA will be heavily limited by infrastructural and economic constraints [56]. While the model here shows increases in cropland in SSA, Mauser et al. [57]found that SSA is a region that could become agriculturally more productive by allocating crops based on profit-maximization and increasing cropping intensity. However, while it may not be possible to expand cropland in SSA, it may be equally unlikely that increased intensification or cropland change is possible, due to similar infrastructural and economic barriers.

Although global cropland area is restricted here to 15% of total ice-free land area, cropland area expansion could, and probably should, be much greater at the individual country scale. The larger conservation of natural land in FC-highNV is facilitated by the slightly stronger intensification and higher yields in FC-highNV compared to FC-lowNV (see Fig 2, 5.2 t ha^-1^ and 4.9 t ha^-1^ in 2050 respectively compared to 3.1 t ha^-1^ in 2000). The difference in global average yield increase, combined with different values for the conservation of natural vegetation lead to different patterns in cropland change at the country scale (Fig 5). For FC-lowNV, low natural vegetation conservation values lead to massive expansion in cropland areas, especially in Central America, North and Central Africa (Libya, Ghana, Niger, and Nigeria) and South-East Asia (Fig 5, panel c). A similar pattern of cropland expansion, though less pronounced compared with FC-lowNV, occurs for FC-highNV (Fig 5, panel d). The increased bioenergy production in FB-highNV causes additional demand for cropland, which leads to larger cropland changes in European countries, South American countries and the US (Fig 5, panel b) compared to FC-highNV (recall, FB-highNV and FC-highNV have the same characteristics, except for bioenergy production).

### 3.3 Further considerations

#### Yield assumptions

PLUM derives crop yields from the LPJ-GUESS by making assumptions about the role of technological development and improved land management [39,47]. There is large potential for modelled yield projections to vary greatly between existing models. This is not unsurprising given that uncertainties can occur in model assumptions regarding, for example, fertiliser effects [58,59], management intensities [60], technology responses [38,61], climate change and the underlying global climate models [62–65]. Despite this however, a recent global gridded model inter-comparison study [65], as part of the Agricultural Model Inter-comparison and Improvement Project (AgMIP), found seven global crop models, including LPJ-GUESS, agreed on the direction of yield changes in response to climate change in major global agricultural regions. Furthermore all models were able to reproduce current relative patterns of crop yields across regions. While CO_2_ fertilisation is unconstrained in this version of LPJ-GUESS, and thus yield projections are high, PLUM counteracted by (1) not accounting for double cropping by deriving global average yield (dividing global production by global cropland area) and (2) including cropland degradation assumptions that result in production losses, decreasing global average yields.

Meeting the food supply and cropland target assumes that the current yield gap (the fraction of how close actual yield is to potential yield, see Licker et al. [66], especially in developing countries, is reduced significantly. In the year 2000, the yield gaps for the countries simulated in PLUM ranged from 0.3 to 0.9, where 0 represents no yield gap [66]. For the model runs that achieve the 2050 food-supply and cropland planetary boundary target, all countries have yield gaps between 0.05 and 0.4. In order to reduce yield gaps to meet food-supply and cropland planetary boundary targets simulations had yield growth rates of 0.9 to 1.0% yr^-1^ over the 2000–2050 period. Other studies have concluded that yield increases of more than 1% per year are needed [38,67], however this often includes increasing yield to meet bioenergy demand in addition to supplying food demand. Furthermore variability exists in what is considered to be an adequate food supply targets (e.g. [38,67]. Compared with the annual yield growth rates of 1.5% yr^-1^ over the 2000–2010 period, and historical yield growth of, for instance, 2.5% yr^-1^ over 1961–1991 (FAOSTAT,[44]), these yield growth rates might appear to be moderate. However, past yield increases are not necessarily a guide to future yields, and future yield potentials are subject to considerable debate [67,68]. For example, socio-economic limitations, the physiological limits to plant growth and climate change have all been suggested as potential limits to further yield growth [69,70].

#### Bioenergy potentials

Comparing bioenergy potential across studies can be misleading because of differences in bioenergy terminology (biomass, bioenergy, biofuels), but also because of different assumptions regarding feedstock (primary crops, residuals from primary crops, residuals forestry or industry, lingo-cellulosic feed-stock) and the availability of technology (first generation vs. second generation, modern bioenergy, conventional or advanced biofuels, traditional biomass). Consequently, within the existing literature there are vast differences in estimates of bioenergy potential [37,38,40,71–75]. Despite this, given the projected bioenergy potential of Haberl et al. [37]of 18 EJ, it might appear surprising that our much lower bioenergy target of 9 EJ by 2050 was not achieved within the planetary boundary of cropland area. Bioenergy potential is very sensitive to yield increases, but the projected crop yield increases used here (accounting for both climate change and CO_2_ fertilization) are similar or even higher (58% and 68% in 2000–2050 for FC-lowNV and FC-highNV respectively) compared to the values used by Haberl et al. [37] in the BAU scenario (54% in 2000–2050). Bioenergy potential is also very sensitive to assumed diets and here the projected food production is considerably higher than that of the Haberl study or the FAO projections. Also, Haberl et al. [37] reported the total calorific value in biomass, whereas we simulate bioenergy as “the energy content in solid, liquid, and gaseous products derived from biomass feed stocks and biogas”[41]. Accounting for this difference, the total calorific value of our bioenergy-mitigation target corresponds to 14 EJ in biomass, which is closer to the estimated potential of 18 EJ by 2050 in Haberl et al. [37].

Projections for both bioenergy demand and food demand vary greatly. For example, Dornburg et al. [71] showed estimates of global demand for biomass in the year 2050 vary between 50 and 250 EJ and similarly, dietary change or lower population growth projections lead to variability in food demand estimates. To account for such uncertainty in demand targets, an alternative approach to the simplicity of setting narrow normative boundaries (as used here), would be to consider the conditions under which simulations fall within a target range. Ultimately, the results presented here depend, on the underlying assumptions about bioenergy (feedstock and available technology) and food demand (diet and population). With different assumptions, such as the use of potentially more efficient second generation bioenergy crops, meeting a bioenergy target might be possible within the planetary boundary of global cropland area. However, while first generation annual crops may currently seem less sustainable than perennial lignocellulose crops, there is still limited experience with second generation bioenergy production and therefore the contribution of first generation bioenergy crops for targets assumed here is not unreasonable.

#### Health and environmental considerations

In general, meeting the food-supply target required a strong increase in meat and milk consumption in emerging and developing countries and thus a continuation/expansion of Western diets. None of our simulations with lower meat consumption met the 2050 food-supply target. However such projected protein-rich, high-fat diets pose potential health threats to countries, arising from obesity and associated poor health [76]. Furthermore, the results show the food surplus currently found in the western world spreads across the globe; indeed obesity has become increasingly wide-spread across the world [77]. Today, almost three times more people are overweight or obese than undernourished [78]. The existence of undernourishment and obesity simultaneously is indicative of the challenge in achieving equality in food security and healthy diets.

Meeting the food supply and bioenergy targets required cropland expansion beyond the cropland planetary boundary and the expansion of cropland into areas of natural vegetation in the tropics found in all scenarios would cause changes in local climate and contribute greatly to emissions from the land use sector [18,79]. We do not consider here targets of sustainability from a biodiversity perspective yet land use change, particularly habitat loss in the tropics, would cause large losses in biodiversity [12,80,81]. Biodiversity has a fundamental role in supporting earth system dynamics and the resilience of a number of planetary boundaries [14,13]. However there is substantial potential for conflict between agricultural land use change, to meet food and bioenergy demand, and conservation targets such as the Aichi Target of expanding the global current protectect areas network cover from 11% to 17% by 2020 [82]. Indeed recent studies have shown both projected land use change and bioenergy production threaten biodiversity because their distribution conflicts with proposed protected areas network expansion [83,84]. Overall, it is unclear whether land sparing and increasing agricultural intensity would be preferable to agricultural expansion with land sharing for biodiversity [81].

An alternative to enhance sustainability in the food sector is the reduction of food waste [85,86]. At the household level alone, 5–21% of food-supply is wasted [45], which provides a large potential to increase food-supply without increasing production. Furthermore, post-harvest losses, which are especially high in countries with poor infrastructure, can contribute to food waste of up to 40% [2,86]. Further research should address the potential of reducing food waste, as well as a sustainable diet in achieving the food-supply target. However, the implementation of demand-side strategies to reduce environmental impact are all connected to highly uncertain processes that are shaped by social behaviour, so supply-side strategies such as sustainable intensification are also needed [87].

## 4. Conclusion

The production of adequate food supply within the cropland planetary boundary under current population trends, economics and diets is only possible with annual crop yield increases of at least 1%. Achieving yield increases requires a reduction in the yield gap in all food producing countries with potential implications for the sustainability of agricultural ecosystems. Achieving bioenergy production from first generation energy crops of an order that would contribute to strong climate mitigation is however not possible at the same time. Yet, recent work indicates that meeting multiple food and energy security goals in a sustainable way could be achieved by a reduction in food waste or the widespread uptake of sustainable diets.

## Supporting information

S1 File Technical documentation for calculation of daily energy supply in PLUM. (DOCX)

S1 Table Parameters kept constant in the simulations and justification for doing so. (DOCX)

S1 Fig Parameter settings for simulation runs that achieve normative targets. Parameter settings for simulations that achieve the food supply target (blue), and simulations that additionally are within the cropland planetary boundary target (yellow), as well as the parameterisation that also achieves the bioenergy target, but is only almost within the cropland planetary boundary (red).
(TIF)

## References

[1] Baldos ULC, Hertel TW. Global food security in 2050: the role of agricultural productivity and climate change. Aust J Agric Resour Econ. 2014;58: 554–570. 10.1111/1467-8489.12048

[2] Godfray HCJ, Beddington JR, Crute IR, Haddad L, Lawrence D, Muir JF, et al Food Security: The Challenge of Feeding 9 Billion People. Science (80-). 2010;327: 812–818. Available: http://science.sciencemag.org/content/327/5967/812.abstract 10.1126/science.1185383 20110467

[3] Lal R. Food security in a changing climate. Ecohydrol Hydrobiol. 2013;13: 8–21. 10.1016/j.ecohyd.2013.03.006

[4] Wiebe K, Lotze-Campen H, Sands R, Tabeau A, van der Mensbrugghe D, Biewald A, et al Climate change impacts on agriculture in 2050 under a range of plausible socioeconomic and emissions scenarios. Environ Res Lett. 2015;10: 85010 10.1088/1748-9326/10/8/085010

[5] Smil V. Worldwide transformation of diets, burdens of meat production and opportunities for novel food proteins. Enzyme Microb Technol. 2002;30: 305–311.

[6] Alexander P, Brown C, Arneth A, Finnigan J, Rounsevell MDA. Human appropriation of land for food: The role of diet. Glob Environ Chang. 2016;41: 88–98. 10.1016/j.gloenvcha.2016.09.005

[7] Kastner T, Rivas MJI, Koch W, Nonhebel S. Global changes in diets and the consequences for land requirements for food. Proc Natl Acad Sci. National Acad Sciences; 2012;109: 6868–6872. 10.1073/pnas.1117054109 22509032 PMC3345016

[8] Tilman D, Balzer C, Hill J, Befort BL. Global food demand and the sustainable intensification of agriculture. Proc Natl Acad Sci. 2011;108: 20260–4. 10.1073/pnas.1116437108 22106295 PMC3250154

[9] FAO/IFAD/WFP. The State of Food Insecurity in the World 2015. Meeting the 2015 international hunger targets: taking stock of uneven progress. Rome, Italy; 2015. 10.3945/an.115.009936 PMC4561839 27352453

[10] UN. Transforming our world: The 2030 Agenda for sustainable development. 2016.

[11] Ramankutty N, Foley JA, Olejniczak NJ. People on the land: Changes in global population and croplands during the 20th century. Ambio. 2002;31: 251–257. 10.1579/0044-7447-31.3.251 12164136

[12] Foley JA, DeFries R, Asner GP, Barford C, Bonan G, Carpenter SR, et al Global consequences of land use. Science (80-). 2005;309: 570–574. 10.1126/science.1111772 16040698

[13] Rockström J, Steffen W, Noone K, Persson Å, Chapin FS, Lambin EF, et al A safe operating space for humanity. Nature. 2009;461: 472–475. 10.1038/461472a 19779433

[14] Rockström J, Steffen W, Noone K, Persson Å, Chapin FS III, Lambin E, et al Planetary boundaries: exploring the safe operating space for humanity. Ecol Soc. 2009;14.

[15] Clarke L, Jiang K, Akimoto K, Babiker M, Blanford G, Fisher-Vanden K, et al Assessing Transformation Pathways. In: Climate Change 2014: Mitigation of Climate Change Contribution of Working Group III to the Fifth Assessment Report of the Intergovernmental Panel on Climate Change. Edenhofer O., Pichs-Madruga R., Sokona Y., Farahani E., Kadner S., Seyboth K., Adler A., Baum I., Brunner S., Eickemeier P., Kriemann B., Savolainen J., Schlömer S., von Stechow TZ C. and JCM, editor. Cambridge UK and New York, NY,USA: Cambridge University Press; 2014.

[16] Luderer G, Pietzcker RC, Bertram C, Kriegler E, Meinshausen M, Edenhofer O. Economic mitigation challenges: how further delay closes the door for achieving climate targets. Environ Res Lett. 2013;8: 34033.

[17] Peters GP, Andrew RM, Boden T, Canadell JG, Ciais P, Le Quéré C, et al The challenge to keep global warming below 2 C. Nat Clim Chang. 2013;3: 4–6.

[18] Engström K, Lindeskog M, Olin S, Hassler J, Smith B. Impacts of climate mitigation strategies in the energy sector on global land use and carbon balance. Earth Syst Dyn Discuss. 2016; 1–43.

[19] Wise M, Calvin K, Thomson A, Clarke L, Bond-Lamberty B, Sands R, et al Implications of limiting CO2 concentrations for land use and energy. Science (80-). 2009;324: 1183–1186. 10.1126/science.1168475 19478180

[20] Azar C, Lindgren K, Obersteiner M, Riahi K, van Vuuren DP, den Elzen KMGJ, et al The feasibility of low CO 2 concentration targets and the role of bio-energy with carbon capture and storage (BECCS). Clim Change. 2010;100: 195–202.

[21] Fuss S, Canadell JG, Peters GP, Tavoni M, Andrew RM, Ciais P, et al Betting on negative emissions. Nat Clim Chang. 2014;4: 850–853.

[22] Van Vuuren DP, Deetman S, van Vliet J, van den Berg M, van Ruijven BJ, Koelbl B. The role of negative CO2 emissions for reaching 2 C—insights from integrated assessment modelling. Clim Change. Springer; 2013;118: 15–27.

[23] FAO. The State of Food and Agriculture: Biofuels: Prospects, Risks and Opportunities. FAO; 2008.

[24] van Vuuren DP, Carter TR. Climate and socio-economic scenarios for climate change research and assessment: Reconciling the new with the old. Clim Change. 2014;122: 415–429. 10.1007/s10584-013-0974-2

[25] O’Neill BC, Kriegler E, Ebi KL, Kemp-Benedict E, Riahi K, Rothman DS, et al The roads ahead: narratives for shared socioeconomic pathways describing world futures in the 21st century. Glob Environ Chang. 2015;

[26] Rounsevell MDA, Metzger MJ. Developing qualitative scenario storylines for environmental change assessment. Wiley Interdiscip Rev Clim Chang. Wiley Online Library; 2010;1: 606–619.

[27] Riahi K, van Vuuren DP, Kriegler E, Edmonds J, O’Neill BC, Fujimori S, et al The Shared Socioeconomic Pathways and their energy, land use, and greenhouse gas emissions implications: An overview. Glob Environ Chang. 2017;42: 153–168. 10.1016/j.gloenvcha.2016.05.009

[28] Popp A, Calvin K, Fujimori S, Havlik P, Humpenöder F, Stehfest E, et al Land-use futures in the shared socio-economic pathways. Glob Environ Chang. 2016;42: 331–345. 10.1016/j.gloenvcha.2016.10.002

[29] Bauer N, Calvin K, Emmerling J, Fricko O, Fujimori S, Hilaire J, et al Shared Socio-Economic Pathways of the Energy Sector—Quantifying the Narratives. Glob Environ Chang. 2015;42: 316–330. 10.1016/j.gloenvcha.2016.07.006

[30] Popp A, Rose SK, Calvin K, Van Vuuren DP, Dietrich JP, Wise M, et al Land-use transition for bioenergy and climate stabilization: Model comparison of drivers, impacts and interactions with other land use based mitigation options. Clim Change. 2014;123: 495–509. 10.1007/s10584-013-0926-x

[31] Von Lampe M, Willenbockel D, Ahammad H, Blanc E, Cai Y, Calvin K, et al Why do global long-term scenarios for agriculture differ? An overview of the AgMIP global economic model intercomparison. Agric Econ. 2014;45: 3–20. 10.1111/agec.12086

[32] Popp A, Lotze-Campen H, Bodirsky B. Food consumption, diet shifts and associated non-CO2 greenhouse gases from agricultural production. Glob Environ Chang. 2010;20: 451–462. 10.1016/j.gloenvcha.2010.02.001

[33] Schmitz C, van Meijl H, Kyle P, Nelson GC, Fujimori S, Gurgel A, et al Land-use change trajectories up to 2050: Insights from a global agro-economic model comparison. Agric Econ. 2014;45: 69–84. 10.1111/agec.12090

[34] Reilly M, Willenbockel D. Managing uncertainty: a review of food system scenario analysis and modelling. Philos Trans R Soc B Biol Sci. 2010;365: 3049–3063. 10.1098/rstb.2010.0141 PMC2935120 20713402

[35] Erb K, Haberl H, Krausmann F, Lauk C, Plutzar C, Steinberger JK, et al. Eating the Planet: Feeding and fuelling the world sustainably, fairly and humanely–a scoping study. Institute of Social Ecology and PIK Potsdam. Vienna: Social Ecology Working Paper No. 116. 2009. doi:ISSN 1726-3816

[36] Erb K-H, Lauk C, Kastner T, Mayer A, Theurl MC, Haberl H. Exploring the biophysical option space for feeding the world without deforestation. Nat Commun. 2016;7. 10.1038/ncomms11382 PMC4838894 27092437

[37] Haberl H, Erb KH, Krausmann F, Bondeau A, Lauk C, Müller C, et al Global bioenergy potentials from agricultural land in 2050: Sensitivity to climate change, diets and yields. Biomass and Bioenergy. 2011;35: 4753–4769. 10.1016/j.biombioe.2011.04.035 22211004 PMC3236288

[38] Lotze-Campen H, Popp A, Beringer T, Müller C, Bondeau A, Rost S, et al Scenarios of global bioenergy production: The trade-offs between agricultural expansion, intensification and trade. Ecol Modell. 2010;221: 2188–2196. 10.1016/j.ecolmodel.2009.10.002

[39] Engström K, Rounsevell MDA, Murray-Rust D, Hardacre C, Alexander P, Cui X, et al Applying Occam’s razor to global agricultural land use change. Environ Model Softw. 2016;75: 212–229. 10.1016/j.envsoft.2015.10.015

[40] Creutzig F, Ravindranath NH, Berndes G, Bolwig S, Bright R, Cherubini F, et al Bioenergy and climate change mitigation: An assessment. GCB Bioenergy. 2015;7: 916–944. 10.1111/gcbb.12205

[41] OECD/IEA. World energy outlook 2012. Paris; 2012.

[42] UN. World Population Prospects: The 2008 Revision. 2009.

[43] FAO. Food security indicators [Internet]. 2016. Available: available: http://www.fao.org/economic/ess/ess-fs/ess-fadata/en.

[44] FAOSTAT. Food and Agriculture Organization of the United Nations: Statistics Division. [Internet]. 2016 [cited 3 Jul 2016]. Available: http://faostat3.fao.org/

[45] Porkka M, Kummu M, Siebert S, Varis O. From food insufficiency towards trade dependency: a historical analysis of global food availability. PLoS One. 2013;8: e82714 10.1371/journal.pone.0082714 24367545 PMC3867377

[46] IPCC. Special report on renewable energy sources and climate change mitigation: summary report for policy makers. [Internet]. 2011. 10.5860/CHOICE.49-6309

[47] Engström K, Olin S, Rounsevell MDA, Brogaard S, Van Vuuren DP, Alexander P, et al Assessing uncertainties in global cropland futures using a conditional probabilistic modelling framework. Earth Syst Dyn. 2016;7: 893–915. 10.5194/esd-7-893-2016

[48] IIASA. SSP Database (version 0.93). International Institue for Applied Systems Analysis, Laxenburg, Austria: International Institute for Applied Systems Analysis, Laxenburg, Austria; 2014.

[49] FAO. Global agriculture towards 2050. High Level Expert Forum—How to Feed the World in 2050. Rome, Italy; 2009.

[50] Alexandratos N, Bruinsma J. World agriculture towards 2030/2050: the 2012 revision. ESA Working paper Rome, FAO; 2012.

[51] Saltelli A, Ratto M, Andres T, Campolongo F, Cariboni J, Gatelli D, et al Global sensitivity analysis: the primer. John Wiley & Sons; 2008.

[52] Smith B, Prentice IC, Sykes MT. Representation of vegetation dynamics in the modelling of terrestrial ecosystems: Comparing two contrasting approaches within European climate space. Glob Ecol Biogeogr. 2001;10: 621–637. 10.1046/j.1466-822X.2001.00256.x

[53] Lindeskog M, Arneth a., Bondeau a., Waha K, Seaquist J, Olin S, et al Implications of accounting for land use in simulations of ecosystem carbon cycling in Africa. Earth Syst Dyn. 2013;4: 385–407. 10.5194/esd-4-385-2013

[54] Masui T, Matsumoto K, Hijioka Y, Kinoshita T, Nozawa T, Ishiwatari S, et al An emission pathway for stabilization at 6 Wm− 2 radiative forcing. Clim Change. 2011;109: 59–76.

[55] van Vuuren DP, Stehfest E, Elzen MGJ, Kram T, Vliet J, Deetman S, et al RCP2. 6: exploring the possibility to keep global mean temperature increase below 2 C. Clim Change. 2011;109: 95–116.

[56] Chamberlin J, Jayne TS, Headey D. Scarcity amidst abundance? Reassessing the potential for cropland expansion in Africa. Food Policy. 2014;48: 51–65. 10.1016/j.foodpol.2014.05.002

[57] Mauser W, Klepper G, Zabel F, Delzeit R, Hank T, Putzenlechner B, et al Global biomass production potentials exceed expected future demand without the need for cropland expansion. Nat Commun. 2015;6. 10.1038/ncomms9946 PMC4660367 26558436

[58] Müller C, Robertson RD. Projecting future crop productivity for global economic modeling. Agric Econ. 2014;45: 37–50. 10.1111/agec.12088

[59] Long SP, Ainsworth EA, Leakey ADB, Nösberger J, Ort DR. Food for Thought: Lower-Than-Expected Crop Yield Stimulation with Rising CO2 Concentrations. Science (80-). 2006;312: 1918–1921. Available: http://science.sciencemag.org/content/312/5782/1918.abstract 10.1126/science.1114722 16809532

[60] Mueller ND, Gerber JS, Johnston M, Ray DK, Ramankutty N, Foley JA. Closing yield gaps through nutrient and water management. Nature. 2012;490: 254–257. Available: 10.1038/nature11420 22932270

[61] Lotze-Campen H, Müller C, Bondeau A, Rost S, Popp A, Lucht W. Global food demand, productivity growth, and the scarcity of land and water resources: A spatially explicit mathematical programming approach. Agric Econ. 2008;39: 325–338. 10.1111/j.1574-0862.2008.00336.x

[62] Ahlström A, Smith B, Lindström J, Rummukainen M, Uvo CB. GCM characteristics explain the majority of uncertainty in projected 21st century terrestrial ecosystem carbon balance. Biogeosciences. 2013;10: 1517–1528. 10.5194/bg-10-1517-2013

[63] Knutti R, Sedláček J. Robustness and uncertainties in the new CMIP5 climate model projections. Nat Clim Chang. 2012;3: 369–373. 10.1038/nclimate1716

[64] Nelson GC, Valin H, Sands RD, Havlík P, Ahammad H, Deryng D, et al Climate change effects on agriculture: economic responses to biophysical shocks. Proc Natl Acad Sci. 2014;111: 3274–9. 10.1073/pnas.1222465110 24344285 PMC3948295

[65] Rosenzweig C, Elliott J, Deryng D, Ruane AC, Müller C, Arneth A, et al Assessing agricultural risks of climate change in the 21st century in a global gridded crop model intercomparison. Proc Natl Acad Sci. 2014;111: 3268–3273. 10.1073/pnas.1222463110 24344314 PMC3948251

[66] Licker R, Johnston M, Foley JA, Barford C, Kucharik CJ, Monfreda C, et al Mind the gap: how do climate and agricultural management explain the “yield gap”of croplands around the world? Glob Ecol Biogeogr. 2010;19: 769–782.

[67] Fischer RA, Byerlee D, Edmeades G. Crop yields and global food security. ACIAR: Canberra, ACT 2014.

[68] Hertel TW. The challenges of sustainably feeding a growing planet. Food Secur. 2015;7: 185–198.

[69] Ray DK, Ramankutty N, Mueller ND, West PC, Foley JA. Recent patterns of crop yield growth and stagnation. Nat Commun. 2012;3: 1293 10.1038/ncomms2296 23250423

[70] Deryng D, Conway D, Ramankutty N, Price J, Warren R. Global crop yield response to extreme heat stress under multiple climate change futures. Environ Res Lett. 2014;9: 34011.

[71] Dornburg V, van Vuuren D, van de Ven G, Langeveld H, Meeusen M, Banse M, et al Bioenergy revisited: Key factors in global potentials of bioenergy. Energy Environ Sci. 2010;3: 258–267. 10.1039/c003390c

[72] Erb KH, Haberl H, Plutzar C. Dependency of global primary bioenergy crop potentials in 2050 on food systems, yields, biodiversity conservation and political stability. Energy Policy. 2012;47: 260–269. 10.1016/j.enpol.2012.04.066 23576836 PMC3617899

[73] Haberl H, Beringer T, Bhattacharya SC, Erb KH, Hoogwijk M. The global technical potential of bio-energy in 2050 considering sustainability constraints. Curr Opin Environ Sustain. 2010;2: 394–403. 10.1016/j.cosust.2010.10.007 24069093 PMC3778854

[74] Smith P, Davis SJ, Creutzig F, Fuss S, Minx J, Gabrielle B, et al Biophysical and economic limits to negative CO2 emissions. Nat Clim Chang. 2016;6: 42–50. Available: 10.1038/nclimate2870

[75] Saura S, Bodin Ö, Fortin M-J. Stepping stones are crucial for species’ long-distance dispersal and range expansion through habitat networks. J Appl Ecol. 2014;51: 171–182. 10.1111/1365-2664.12179

[76] WHO. Fact sheet N 311: Obesity and overweight [Internet]. 2016 [cited 8 Aug 2016]. Available: http://www.who.int/mediacentre/factsheets/fs311/en/

[77] Tilman D, Clark M. Global diets link environmental sustainability and human health. Nature. 2014;515: 518–522. http://www.nature.com/nature/journal/v515/n7528/full/nature13959.html 10.1038/nature13959 25383533

[78] NCD-RisC. Trends in adult body-mass index in 200 countries from 1975 to 2014: a pooled analysis of 1698 population-based measurement studies with 19.2 million participants. Lancet. 2017;387: 1377–1396. 10.1016/S0140-6736(16)30054-X PMC7615134 27115820

[79] Lawrence D, Vandecar K. Effects of tropical deforestation on climate and agriculture. Nat Clim Chang. Nature Research; 2015;5: 27–36.

[80] Sala OE, Chapin FS, Armesto JJ, Berlow E, Bloomfield J, Dirzo R, et al Global biodiversity scenarios for the year 2100. Science (80-). 2000;287: 1770–1774. 10.1126/science.287.5459.1770 10710299

[81] Phalan B, Onial M, Balmford A, Green RE. Reconciling food production and biodiversity conservation: land sharing and land sparing compared. Science (80-). 2011;333: 1289–1291. 10.1126/science.1208742 21885781

[82] Secretariat of the Convention on Biological Diversity. Global Biodiversity Outlook 4. Montreal, Canada; 2014.

[83] Santangeli A, Toivonen T, Pouzols FM, Pogson M, Hastings A, Smith P, et al Global change synergies and tradeoffs between renewable energy and biodiversity. Gcb Bioenergy. 2016;8: 941–951.

[84] Pouzols FM, Toivonen T, Di Minin E, Kukkala AS, Kullberg P, Kuustera J, et al Global protected area expansion is compromised by projected land-use and parochialism. Nature. 2014;516: 383–386. Available: 10.1038/nature14032 25494203

[85] Parfitt J, Barthel M, Macnaughton S. Food waste within food supply chains: quantification and potential for change to 2050. Philos Trans R Soc B Biol Sci. 2010;365: 3065–3081. 10.1098/rstb.2010.0126 PMC2935112 20713403

[86] Alexander P, Brown C, Arneth A, Finnigan J, Moran D, Rounsevell MDA. Losses, inefficiencies and waste in the global food system. Agric Syst. 2017;153: 190–200. 10.1016/j.agsy.2017.01.014 28579671 PMC5437836

[87] Smith P, Haberl H, Popp A, Erb K-H, Lauk C, Harper R, et al How much land-based greenhouse gas mitigation can be achieved without compromising food security and environmental goals? Glob Chang Biol. 2013;19: 2285–302. 10.1111/gcb.12160 23505220

