# Supplementary material for: Food supply and bioenergy production within the global cropland planetary boundary
Source: PLoS One. 2018 Mar 22;13(3):e0194695. doi: 10.1371/journal.pone.0194695 (PMC5864037; doi:10.1371/journal.pone.0194695)
Supplement: S1 File — (DOCX) [file pone.0194695.s001.docx]

For the calculation of daily energy supply we assumed that the energy content of modelled food commodities (cereals, milk, and meat) will not change (constant at year 2000 values). Country-specific energy contents for cereals, milk and meat (*e_Creals_, e_Milk_, e_Meat_*) were derived from (1).

$e_{x, c}=\frac{{kcal}_{x, c,i}*365}{C{onPc}_{x,c,i}}$ (1)

where *x*=Cereals, Milk, Meat, *c*=countries, *i*=initial year (here 2000) and *kcal* = energy supplied by item *x* in country *c* in 2000 (kcal cap^-1^ d^-1^) and *ConPc_i_* = consumption of item *x* for country *c* in 2000 (kg cap^‑1^ yr^‑1^).

The energy content for each food commodity (kcal_Cereals_, kcal_Milk_, kcal_Meat_) was then multiplied with the projected changes in food demand, see (2).

${kcal}_{x, c,t}={ConPc}_{x,c,t}*e_{x,c}$ (2)

where *ConPc_t_* is the projected per capita food demand for each food item (kg cap^‑1^ yr^‑1^).

In cases where data were incomplete and did not allow the calculation of country-specific energy contents, the average energy contents for cereals (3026 kcal kg^‑1^), meat (1806 kcal kg^‑1^) and milk (602 kcal kg^‑1^) were used.

The sum of the energy provided by cereals, milk and meat was calculated (*kcalPcPLUM*). To estimate the total supply of energy, *kcalPcPLUM* was scaled to match total per capita energy supply reported in FAOSTAT in 2000 with the scaling factor *kcalRatio_i*. The scaling factor *kcalRatio_i* could not be derived for the Democratic Republic of Congo, Burundi, Comoros, Eritrea, Palestine, Syria and Libya, because of missing data. Instead, we assumed *kcalRatio_i* close to neighbouring countries or countries with similar per capita energy supply. This scaling was applied throughout the simulation period. However, some countries can have very high *kcalRatio_i*, as a large share of their diet is provided by food commodities (i.e. roots and tubers) other than the three commodities included here. Therefore, if for these countries (*kcalRatio_i* > 2.5) per capita meat and milk consumption increases in the simulations, *kcalRatio_i* is adjusted downward to account for the situation that increasing consumption of milk and meat production reduces the consumption of other food commodities. To derive scaling factors, linear regression was performed for all countries with *kcalRatio_i*>= 2.5 with per capita meat consumption (*meatPc*) as independent variable.
