# Supplementary material for: Food supply and bioenergy production within the global cropland planetary boundary
Source: PLoS One. 2018 Mar 22;13(3):e0194695. doi: 10.1371/journal.pone.0194695 (PMC5864037; doi:10.1371/journal.pone.0194695)
Supplement: S1 Table — (DOCX) [file pone.0194695.s002.docx]

| **Parameter (*unit*)** | **Description** | **PLUM Parameter settings that reproduce average current trends (2000-2010)** | **Justification to keep parameter constant at current trend value in simulations** |
| --- | --- | --- | --- |
| gdp | economic income of a country | 0 | +/-5 of global aggregated GDP in 2050 with varied impact on countries, deterministic GDP projections used in simulations |
| pop | population size of a country | 0 | +/- 1% of global population in 2050, deterministic population projections used in simulations |
| overProd Rate *(1/time)* | Global parameter representing cereal produced without specific purpose (e.g. harvest buffer) | 0 | importance < 0.1 in Global Sensitivity Analysis (GSA; Engström et al., 2016b) |
| cerealVar *(1/time)* | global parameter controlling variability of cereal consumption | 0 | importance < 0.1 in GSA (Engström et al., 2016b) |
| meat 2 *(kg meat per capita/ log(GDP per capita))* | Rate of increase in meat consumption with increasing GDP. *Group 2 applies to high income countries with a low per capita consumption of animal products* | 35 | classes meat2 and milk2 only represent <10 countries and these classes was therefore excluded here, instead most meat2 countries belong now to class meat3 and milk2 countries belong to class milk1 and class milk3 |
| milk 2 (*kg meat per capita/ log(GDP per capita))* | Rate of increase in milk consumption with increasing GDP. | 40 |  |
| distribution *(1/time)* | describes the distribution of technology within a country, negatively correlated to the percentage of rural population | 1 | negligible impact compared to technology and investments (global average yield for min 5.11 and max 5.13 ton ha^‑1^) |
| abandonCL *(unitless)* | Abandonment rate of cropland in developed countries | 0.05 | The value for the parameters are maximum values, i.e. they only restrict the internally calculated land conversion rates if these exceed the values for the parameter. Very limited implication at the global scale (importance < 0.1 in GSA performed in Engström et al., 2016b). However, in specific cases (drastic cropland in- or decrease) these parameters can have implications for single countries. |
| abandonCL_D *(unitless)* | Abandonment rate of cropland in developing countries | 0.05 |  |
| newCL *(unitless)* | Conversion rate of new cropland in developed countries | 0.05 |  |
| newCL_D (*unitless*) | Conversion rate of new cropland in developing countries | 0.05 |  |
| newCLs (*unitless*) | Conversion rate of new cropland in developed countries for self sufficiency | 0 |  |
| newCLs_D (*unitless*) | Conversion rate of new cropland in developing countries for self sufficiency | 0.02 |  |
| grassForest *(%)* | Global parameter, ratio of cropland converted to or from grasslands and forests | 0.5 | Affect only the distribution of abandoned cropland/forest and grassland decrease (grassForest) as well as the natural degradation of forest (forestDeg). Grassland and forest are not analysed for simulations, so not relevant here. |
| forestDeg *(1/time)* | Rate of forest degradation | 0 |  |
