# Supplementary figures and images for: Food supply and bioenergy production within the global cropland planetary boundary
Source: PLoS One. 2018 Mar 22;13(3):e0194695. doi: 10.1371/journal.pone.0194695 (PMC5864037; doi:10.1371/journal.pone.0194695)

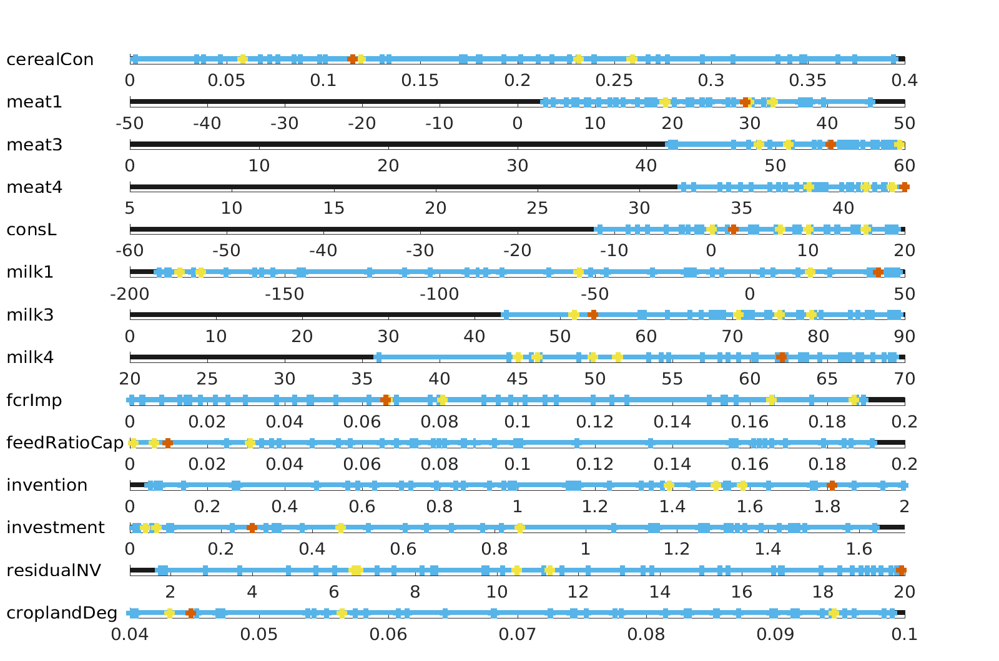

Supplement: S1 Fig — Parameter settings for simulations that achieve the food supply target (blue), and simulations that additionally are within the cropland planetary boundary target (yellow), as well as the parameterisation that also achieves the bioenergy target, but is only almost within the cropland planetary boundary (red). (TIF) [file pone.0194695.s003.tif]
